# Radiological and Pulmonary Results of Surgical Treatment of Severe Idiopathic Scoliosis Using Preoperative Halo Gravity Traction Compared with Less Invasive Temporary Internal Distraction in Staged Surgery in Adolescents

**DOI:** 10.3390/jcm13102875

**Published:** 2024-05-13

**Authors:** Pawel Grabala, Michael A. Galgano, Michal Grabala, Jacob M. Buchowski

**Affiliations:** 1 Department of Pediatric Orthopedic Surgery and Traumatology, Medical University of Bialystok and Medical University of Bialystok Children’s Clinical Hospital, ul. Waszyngtona 17, 15-274 Bialystok, Poland; 2 Paley European Institute, Al. Rzeczypospolitej 1, 02-972 Warsaw, Poland; 3 Department of Neurosurgery with Department of Interventional Neurology, Medical University of Bialystok and Medical University of Bialystok Clinical Hospital, ul. M. Sklodowskiej-Curie 24A, 15-276 Bialystok, Poland; 4 Department of Neurosurgery, University of North Carolina, Chapel Hill, NC 27516, USA; mgalgano@email.unc.edu; 5 2nd Clinical Department of General and Gastroenterogical Surgery, Medical University of Bialystok, ul. M. Skłodowskiej-Curie 24a, 15-276 Bialystok, Poland; michal@grabala.pl; 6 Department of Orthopedic Surgery, Washington University School of Medicine, 660 S Euclid Ave., St. Louis, MO 63110, USA; buchowskij@wustl.edu

**Keywords:** severe scoliosis, Halo, HGT, less invasive temporary internal distraction, scoliosis, spinal deformity, temporary internal distraction, neglected scoliosis, Halo gravity traction

## Abstract

**Background:** Severe and rigid scoliosis represents a type of spinal deformity characterized by a Cobb angle exceeding 90° and a flexibility of less than 30%. Halo spinal traction remains the established standard for managing severe scoliosis, although alternative approaches such as temporary internal distraction rods and staged surgical correction exist. The primary objective of this investigation was to compare two cohorts of patients treated using these distinct methods to ascertain any divergences in terms of surgical and radiological outcomes, pulmonary function (PF), and quality of life (QoL). **Methods**: This study encompassed a total of 62 pediatric patients meeting the specified criteria, which included severe idiopathic scoliosis (major Cobb curve >90) and flexibility <30%. Group 1 (G1) underwent surgical intervention involving preoperative Halo gravity traction (HGT) succeeded by posterior spinal fusion (PSF). On the other hand, Group 2 (G2) underwent a two-stage procedure starting with a less invasive temporary internal distraction technique (LITID) prior to PSF. The radiological outcomes, PF, and QoL were documented and assessed over a monitoring period ranging from 2 to 5 years. **Results:** The average preoperative major curves (MCs) measured 124° and 122° in G1 and G2, respectively (*p* < 0.426). Initial flexibility, as observed in preoperative bending films, ranged from 18% in G1 to 21% in G2 (*p* < 0.001). Following the ultimate surgical intervention, the MCs were corrected to 45° and 37.4° in G1 and G2, respectively (*p* < 0.001). The percentage correction of the MCs was higher in G2 (63% vs. 70% in G1 and G2, respectively), with significant between-group disparities (*p* < 0.001). The mean preoperative thoracic kyphoses (TKs) were 96.5° in G1 and 92° in G2 (*p* = 0.782), which were rectified to 45.8° in G1 and 36.2° in G2 (*p* < 0.001), equating to correction rates of 55% and 60% in the respective groups. Initially, G2 exhibited lower values for the percentage of predicted lung volume (FVC) and predicted FEV1 compared with G1 (49% and 58% vs. 54.5% and 60.8%; N.S.). Nonetheless, both groups demonstrated enhancements in their FVC and FEV1 values over the follow-up period. **Conclusions:** The surgical management of severe and untreated spinal curvatures in the pediatric and adolescent population can be considered safe, with a tolerable incidence of minor complications. LITID emerges as a method offering improved QoL and pulmonary function, achieving notably substantial average corrections in deformity by 70% in the coronal plane and 60% in the sagittal plane, alongside a mean increase in trunk height of 10.8 cm. Furthermore, a typical reduction of 76% in rib humps and enhancements in respiratory function, as indicated by improvements in 1 s predicted forced expiratory volume (by 25–56%) and forced vital capacity (by 35–65%), were achieved, leading to a clinically and statistically significant enhancement in QoL when evaluated using SRS-22r, without resorting to more radical, high-risk procedures.

## 1. Introduction

Severe and rigid scoliosis is a form of spinal deformity in which the Cobb angle of the main curvature is greater than 90° and flexibility is less than 30% [1,2]. Severe and untreated scoliosis can have a serious impact on a patient’s health and quality of life, and the surgical correction of large curves is difficult because it is associated with a high risk of neurological injury, especially when a large curve is attempted in one procedure [3,4,5,6,7]. Additionally, if left untreated and still progressing, serious deformities of the spine can lead to dysfunction of the entire body, causing disorders of the nervous and circulatory–respiratory systems, which, in turn, affect the growth, appearance, and long-term mortality of patients [7,8].

The preferred treatment approach for severe and rigid spinal curvatures is the utilization of preoperative Halo gravity traction (HGT) [9,10,11,12,13,14,15]. However, various alternative methods have also been introduced for managing severe spinal deformities, such as temporary internal distraction (TID) and its various adaptations, anterior release (AR), posterior release (PR), and thoracoplasty with rib osteotomy (TP), as well as different forms of three-column osteotomies like pedicle subtraction osteotomy (PSO) and vertebrectomy (commonly known as vertebral column resection (VCR)) [16,17,18,19,20,21,22,23,24,25]. Techniques involving gradual traction, whether external or internal, are typically viewed as safer compared with the more aggressive three-column osteotomy (3CO) approach, with a confined and acceptable incidence of complications [3,4,6,10,11,12,15,16,17,20,24,26].

There is a scarcity of studies within the medical literature that directly compare both methodologies for managing severe spinal deformities in the pediatric and adolescent populations, specifically the HGT strategy versus the less invasive temporary internal distraction rod technique [20,26]. Thus, the objective of this investigation was to assess two cohorts of patients undergoing treatment with these respective techniques to more accurately discern the variances in terms of surgical and radiological results, pulmonary function (PF), and quality of life (QoL).

## 2. Materials and Methods

### 2.1. Setting and Patients

In this retrospective analysis, 62 pediatric individuals diagnosed with severe idiopathic scoliosis (defined as a major Cobb curve exceeding 90 degrees) and flexibility below 30% were enrolled for surgical intervention: either preoperative Halo gravity traction (HGT) followed by posterior spinal fusion (PSF) or a two-step procedure commencing with a minimally invasive temporary internal distraction technique (LITID) in the first phase and concluding with PSF in the second phase. Evaluation of the treatment efficacy, particularly in terms of spinal deformity correction, encompassed measurements of pulmonary function (PF) and quality of life (QoL). The follow-up duration ranged from 2 to 5 years, with a minimum of 2 years. Only patients with a follow-up period longer than 2 years were included in the study. The maximum follow-up period of our patients when treated with the LITID technique is 5 years. All patients after surgical treatment are monitored every 3 months 1 year after surgery and then every 6 months 1 year after surgery. The surgical procedures were conducted between 2016 and 2022 at a pediatric orthopedic spine center within a children’s hospital by a consistently experienced orthopedic spine surgeon specializing in pediatric cases. The patients were presented with two treatment choices: HGT or LITID. Not all patients favored HGT, and similarly, not all opted for the staged approach involving LITID. The ultimate decision rested upon discussions involving the patient, parents, and healthcare provider, considering the entirety of the treatment process, potential complications, and anticipated advantages. All patients in this study received previous conservative treatment that included intensive rehabilitation and physiotherapy, and braces or casts were used. The patients who underwent preoperative HGT constituted Group 1 (*n* = 20), while Group 2 (*n* = 42) comprised individuals treated with LITID and staged surgery, subsequently followed by PSF. All participants and their legal guardians provided written informed consent for study involvement and the dissemination of findings, with approval obtained from the ethics committee of the district hospital (APK.002.80.2020).

### 2.2. Clinical, Radiological, and Functional Measures

All patients meeting the criteria for surgical intervention of spinal deformities underwent preoperative magnetic resonance imaging (MRI) of the entire spine to identify or rule out additional pathologies affecting the spinal cord. In this investigation, fundamental chest measurements, radiographic assessments of spinal curvatures, pulmonary function (PF), and quality of life (QoL) parameters were documented for all participants enrolled. Specific parameters related to spinal deformities were documented and scrutinized, including the Cobb angle of the upper thoracic curvature, primary thoracic and lumbar curvature, and measurements of the sagittal plane—thoracic kyphosis (T5–T12) and lumbar lordosis (T12–S1). Furthermore, spinal flexibility was documented and assessed through flexion and extension radiographs. An evaluation was also conducted on rib prominence measurements, trunk height, and apical vertebral translation (AVT). The findings from the pulmonary function tests—forced vital capacity (FVC) and forced expiratory volume in 1 s (FEV1) as a percentage of predicted values—and any perioperative complications were meticulously documented. Quality of life assessments were gauged utilizing the Scoliosis Research Society-22 revised (SRS-22r) questionnaire. All acquired and documented parameters were evaluated preoperatively, post-initial-surgery, following definitive surgery and posterior spinal fusion (PSF) and during the ultimate follow-up phase. Radiographic parameters were assessed by an impartial evaluator. The surgical procedures were executed under the guidance of intraoperative spinal cord monitoring: somatosensory-evoked potentials (SSEPs) and transcranial motor-evoked potentials (MEPs) [17].

### 2.3. Statistical Analysis

Statistical analysis software was utilized for the computations and to conduct all analyses (version 10.0; StatSoft Inc., Tulsa, OK, USA). To handle and present the gathered data, the standard deviation (SD) of the mean, 95% confidence interval (CI), medians with lower and upper quartiles, and frequency were employed. The Shapiro–Wilk test, the Mann–Whitney U test, and the Kruskal–Wallis analysis were also incorporated. Both parametric and non-parametric methodologies were executed to compare the obtained results within both cohorts. The ANOVA test, along with the Tukey–Kramer method, was also implemented. Pearson correlation coefficients were computed to assess the association between two numerical variables. Alterations between the two time points were evaluated through McNemar tests. A significance level of *p* < 0.05 was deemed statistically noteworthy.

### 2.4. Surgical Technique

**Figure 1:**
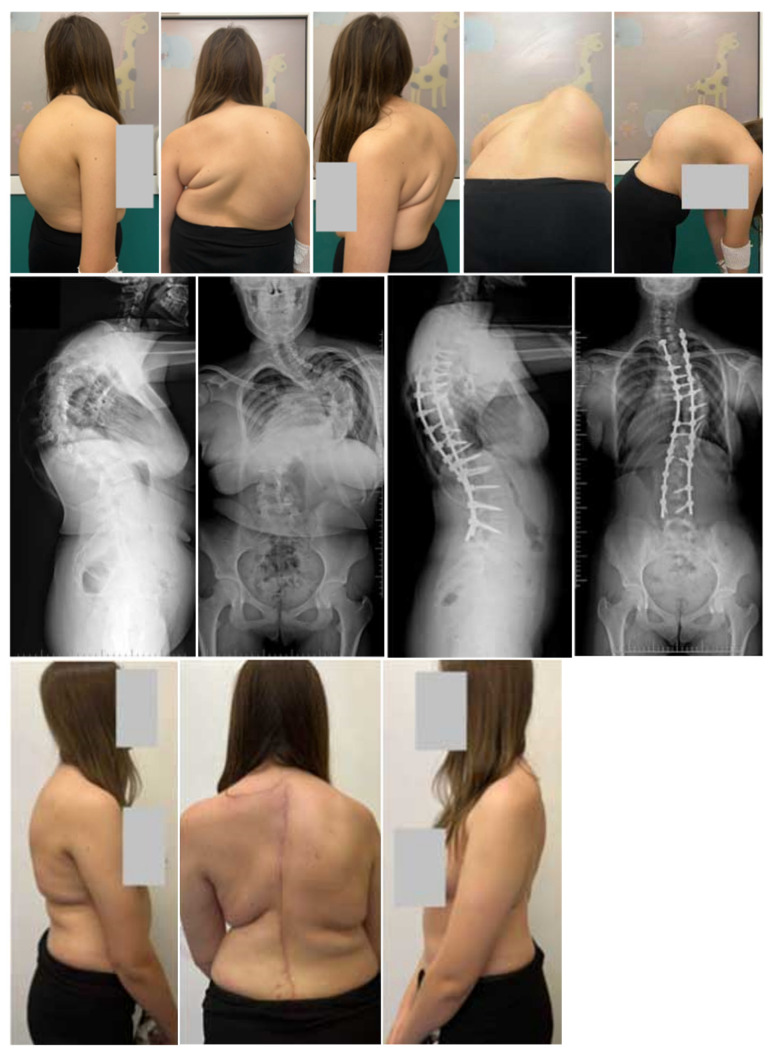
A 14-year-old female patient treated with preoperative HGT followed by posterior column osteotomies and PSF. Clinical photographs and radiographs performed pre- and postoperatively at final follow-up.

Patients allocated to G1 underwent preoperative Halo gravity traction (HGT). The surgical procedure entailed the insertion of a Halo ring under general anesthesia, following the methodology outlined in existing studies [10,13,15]. This involved utilizing 8–10 pins to optimize distraction, thereby decreasing the likelihood of pin pullout and instability in the Halo ring during traction. Traction initiation commenced at 2–3 kg and was incrementally augmented to a maximum of 50% of the patient’s body weight, mirroring protocols detailed in prior studies [4,10,20]. Subsequent to the completion of the HGT regimen, corrective measures for the spinal deformity were undertaken through a conventional posterior approach employing segmental screw instrumentation via a free-hand technique, alongside posterior column osteotomy, as documented in the pertinent literature [27,28,29,30,31]. A case illustration showcasing the management of severe idiopathic scoliosis with HGT and posterior spinal fusion (PSF) is delineated in Figure 1.

**Figure 2:**
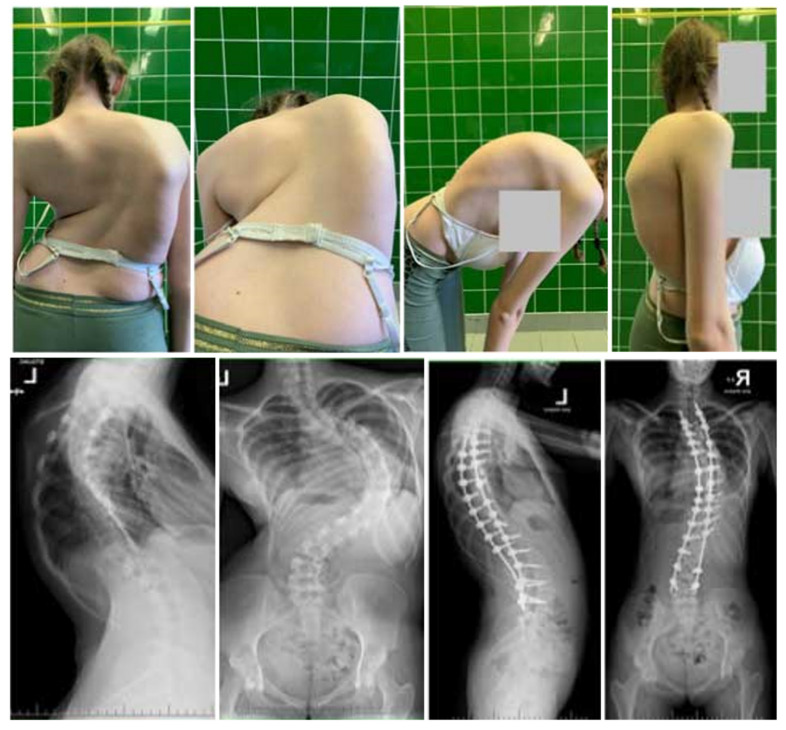

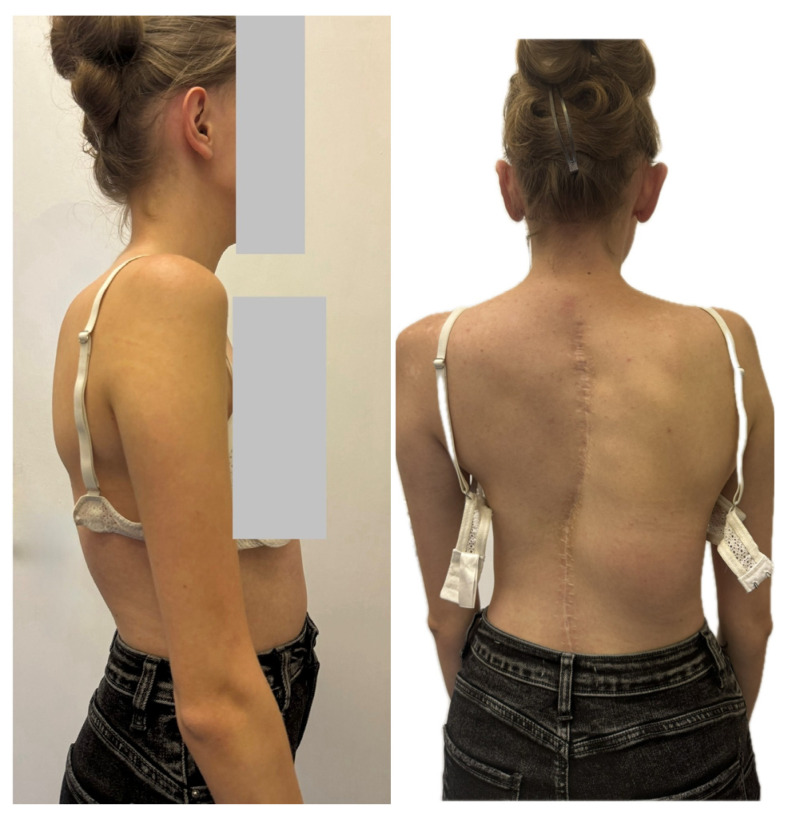
A 17-year-old female patient treated with staged surgery with LITID followed by PSF. Clinical photographs and radiographs performed pre- and postoperatively at final follow-up.

The surgical intervention for patients in the G2 cohort was bifurcated into two stages. A refined version of the internal distraction techniques elucidated by Buchowski et al. [16] and Skaggs et al. [17], extensively expounded upon by Grabala and Helenius [24], was implemented during the surgical intervention. In essence, during the initial procedure, akin to minimally invasive surgical strategies for the standard deployment of growing rods across the proximal thoracic and lower lumbar spinal levels, segmental screws were situated, accompanied by posterior column osteotomies, typically within the exposed upper and lower spinal regions. Subsequently, two titanium alloy rods (primarily with a 6.0 mm diameter) were subcutaneously positioned and interconnected to the segmental screws, facilitating synchronous initial correction under neuromonitoring (NM). Furthermore, the precise internal distraction of the deformed spine was achieved with the aid of NM. The definitive surgery was scheduled 2–6 weeks after the initial operation and encompassed posterior column osteotomies (PCOs) at the deformity apex, bilateral segmental pedicle screw placement, and ultimate correction utilizing two contoured 6.0 mm cobalt–chromium rods [27]. The curvature correction entailed a combination of techniques such as compression–distraction, rod cantilevering, and derotation. An example of a patient managed with lengthening instrumentation for the treatment of deformity (LITD) is presented in Figure 2.

## 3. Results

### 3.1. Clinical Characteristics and Radiographic and Functional Outcomes

A total of 50 females and 12 males diagnosed with severe idiopathic scoliosis were the subjects of this study. Group 1 (G1) consisted of 20 individuals (18 females and 2 males) with an average (SD) age of 16.5 (3.5) years, while Group 2 (G2) included 42 patients (32 females and 10 males) with an average (SD) age of 16.4 (4.8) years, as detailed in Table 1. The initial mean (SD) preoperative main curvatures (MCs) measured 124° (10.8) for G1 and 122° (9.8) for G2, with no significant difference observed (*p* < 0.426). The preoperative flexibility (SD), as indicated by bending films, ranged from 18% (7.2) in G1 to 21% (9.5) in G2, showing a significant variation (*p* < 0.001). Following the final surgery, the corrected MCs (SD) were 45° (13.8) for G1 and 37.4° (11.4) for G2, with a notable difference (*p* < 0.001). The percentage correction of the MCs was notably higher in G2 compared with G1 (63% vs. 70%), with statistical significance (*p* < 0.001). No progression of MCs was evident during the follow-up in either group, as depicted in Table 2. Initially, the mean thoracic kyphoses (SD) were 96.5° (14.5) for G1 and 92° (11.8) for G2, showing no significant variation (*p* = 0.782). Post-surgery, the corrected values were 45.8° (15.8) for G1 and 36.2° (10.2) for G2, displaying a significant difference (*p* < 0.001). The mean preoperative lumbar lordoses (SD) measured at −60.6° (23.6) for G1 and −46° (10.8) for G2 and were corrected to −41° (12.9) for G1 and −36.4° (10.6) for G2 (*p* = 0.001). The initial anterior vertebral translation (AVT) values (SD) improved from 78 mm (21.8) to 35 mm (14.6) in G1 and from 73.2 mm (21.5) to 28.2 mm (18.4) in G2 at the last follow-up (*p* < 0.393). Trunk height mean (SD) also showed an increase post-surgery from 28.8 (7.1) cm to 36.5 (6.8) cm in G1 and from 29.2 (4.2) cm to 38.3 (2.8) cm in G2, indicating a significant difference between the two groups (*p* < 0.001). No significant divergence was observed at the final follow-up. Initially, the predicted lung volume (FVC) percentage was lower in G2 compared with G1 (49% vs. 54.5%; *p* < 0.132). Similarly, the predicted FEV1 value percentage was lower in G2 than in G1 (58% vs. 60.8%; *p* < 0.221). Over the 2-year follow-up period, the predicted FVC percentage significantly improved in both G1 and G2 (*p* < 0.001). Furthermore, notable enhancements were observed in the predicted FEV1 values during the follow-up in both G1 and G2 (*p* < 0.001), with no statistical discrepancies observed throughout the follow-up period (Table 2). There was no significant loss of correction with time. Representative cases are shown in Figure 1 (HGT and PSF) and Figure 2 (TID and PSF). Detailed results of all radiographic measures are summarized in Table 2 and Table 3.

The mean preoperative SRS-22r total score improved (SD) significantly from 2.88 (0.92) to 4.33 (0.65) in Group 1 and from 3.22 (0.42) to 4.46 (0.45) in Group 2 during the follow-up period (*p* < 0.001 for both comparisons; Table 4). No statistical difference was found in the SRS-22r score between the groups at the time of the final follow-up.

### 3.2. Complications

Complications were observed in both cohorts of patients under investigation. The overall incidence of complications was greater in Group 1 (140%) compared with Group 2 (48%), displaying a statistically significant variance (<0.001). Worth highlighting, though, is that individual patients could experience multiple complications. Noteworthy alterations in neuromonitoring were identified during the concluding phases of the surgical procedures, as well as the final adjustment and posterior spinal fusion (PSF), manifesting in 15% of Group 1 patients and 12% of Group 2 patients (NS). These neuromonitoring changes were linked to the corrective distraction of the deformed spinal column. Upon a reduction in spinal distraction, the neuromonitoring responses reverted to their baseline levels. No fresh postoperative neurological impairments were detected in either group. Throughout the therapeutic regimen in both cohorts, encompassing the period between the initial placement of HGT or LITID and the definitive surgery, instances of neck and back discomfort were documented in both groups (<0.001), with a notably higher frequency in Group 1 (55%), as delineated in Table 5. No untoward events were noted during the ultimate follow-up evaluation. Also noteworthy is that none of the subjects needed any specialized interventions.

## 4. Discussion

The idea of introducing a temporary internal distraction was proposed to obtain effects in the treatment of severe and rigid spinal deformity comparable to those obtained through the use of preoperative Halo traction with the lowest possible burden on the patient’s body and by minimizing possible complications [10,15,25]. One of the main advantages of HGT is the simplicity of this method and the minimal invasiveness of the required surgery, and thanks to this, the use of slow and gradual gravitational traction provides safe corrections in curvature. As a result, less load is exerted during the final corrective surgery using transpedicular screws and rods due to the previously achieved correction. Although HGT is widely used for the treatment of large spinal deformities in children and adolescents, unfortunately, some limitations to its use still exist, and its use may even be completely contraindicated in some patients, such as those with congenital defects of the occipital–cervical joint and instability in the cervical spine [13,25,32,33].

HGT necessitates a temporary and gradually increasing application of force, and it is linked to a prolonged hospitalization period. Based on the existing literature, preoperative HGT may offer distinct advantages in managing severe spinal deformities, enabling corrections of 35% in the frontal Cobb angle and 35% in the sagittal Cobb angle in certain cases. Additionally, it aids in enhancing lung function by 9% [10,15,32,34,35], an outcome that aligns with those observed in our study’s Group 1 patients. A notable benefit of HGT therapy is its cost-effectiveness, requiring fewer financial investments compared with implant utilization or the application of magnetically controlled growing rods for internal temporary traction, as discussed in various studies [11,12,13,14,20]. Nevertheless, a direct comparison of overall treatment expenses is challenging due to variations influenced by a country’s healthcare system and insurance framework. McIntosh et al. examined individuals undergoing preoperative HGT and determined that employing this method led to trunk elongation and decreased friction amid the ribs and pelvis. Moreover, rib expansion within a concave region facilitates diaphragm displacement, reducing respiratory restrictions [10,32,35].

In our study, trunk height increased by a mean value of 8 cm during HGT. Additionally, the literature also describes improvements in the nutritional status and lung function of patients after treatment with preoperative HGT [15]. One of the latest systematic reviews showed that the use of preoperative HGT in cases of spine deformities effectively reduces scoliotic and kyphotic curvatures and, at the same time, reduces the number of complications in subsequent spinal fusion surgery [34]. Regarding the weight used for traction, the available research shows uniformity, ranging from 30 to 50% of the total body weight. In a special way, slow, gradual traction prepares the spinal cord for final correction. This action leads to the release of the apical tether located in the spinal cord. The reduction in the curvature partially alleviates the burden placed on the spinal cord according to previous research [25,36]. A study conducted by Yang and colleagues [37] demonstrated that preoperative traction has the potential to streamline the surgical process by decreasing deformity and enhancing the cardiopulmonary function of patients. Additionally, it facilitates the assessment of spinal cord tolerance and diminishes the likelihood of spinal cord and nerve damage. Koller et al. reported an enhancement in neurological function before surgery in five patients experiencing progressive spasticity at the onset of Halo gravity traction [25]. Despite numerous studies on Halo gravity traction (HGT), no unanimous agreement on the most effective traction regimen or the utilization of Halo in clinical settings exists yet. In a separate investigation [38], the majority of corrections were observed to take place during the initial 2 weeks of HGT. The researchers concluded that determining the ideal duration of traction should involve an evaluation of potential complications. Nemani and colleagues [8] proposed that the correction of deformities using HGT is rapid initially but eventually plateaus around 63 days. However, no definitive recommendation on the optimal duration for maintaining patients under traction exists yet, and whether the plateau was due to reduced patient compliance with HGT, extended periods without traction, or an increase in patient weight leading to a decrease in the traction-weight-to-patient-weight ratio remains uncertain. In our experience, HGT can be performed at home for some patients, but this requires the adaptation of the equipment and daily rehabilitation with physiotherapy at home. Such a patient must be in constant contact with the attending physician due to the risk of complications related to the Halo ring and stretching, among others (e.g., cranial nerve injuries, pin loosening, or pin infections) [10,13,34]. In a study conducted by Popescu et al. [39], complications associated with traction therapy were observed to affect 94.7% of patients, with the most prevalent being cervical pain occurring in 89.5% of cases and back pain in 36.8%. Group 1 exhibited similar complications, at a rate of 55%. The authors of [39] also identified pin-related issues, though only in 5.3% of cases, with symptoms such as vertigo and pin displacement [39]. The researchers also documented neurological manifestations in 26.3% of patients, along with pin discomfort and infection impacting another 26.3% of individuals. Within our investigation, 35% of pin infections were found among patients undergoing HGT. Despite these minor setbacks being commonly linked to HGT, diligent patient monitoring can effectively address them, thus rendering traction a secure technique for gradual curvature correction. Furthermore, the complications are generally well tolerated by patients [39].

As an alternative to external traction techniques that use Halo traction, we can use internal traction—the TID method—in various configurations [16,19,20,24,40,41,42]. We found that one study compared the outcomes of a Halo gravity traction course with temporary internal distraction [26]. The authors analyzed 19 patients with severe scoliosis with various etiologies: 7 treated with temporary internal distraction and 12 treated with Halo gravity traction. However, the used surgical technique for TID was different from the one we used. The authors performed a wide posterior approach with segmental screw placement; the second stage was performed after 15 days; during the period between the surgeries, the patients could not walk and were restricted to bed. In that study [26], the authors also did not note whether the intergroup differences were significant to establish, which is the most effective method. By contrast, our LITID technique was shown to allow for the elimination of long-term hospital treatment and the application of a greater traction force to the spine when stretching it after applying posterior release (PCO), which provides strong distraction mechanisms to the deformed spine [16,24,40]. Of course, nothing is perfect, and this technique is not acceptable for everyone due to the need for staged surgery as part of the treatment course, i.e., two surgeries under anesthesia and a stay in the operating theater. However, as a result of being conducted as a staged procedure, temporary internal distraction (TID) enables a precise evaluation of neurological function in a conscious and mobile patient. TID proves to be more beneficial for treating severe scoliotic multi-segment deformities in contrast to short rigid curves, which are more suitable for osteotomies [19,43]. Similarly, the outcomes presented by Buchowski et al. [16] considered the utilization of temporary internal distraction rods in staged surgery. The primary curve correction following the initial posterior release, along with internal distraction, resulted in an average correction of 53%, while the final main curve correction averaged 80% [16]. By modifying Buchowski’s technique [20], we were able to employ a less invasive temporary internal distraction in this study, resulting in an average correction of 70% for the main coronal curvature and 60% for thoracic kyphosis during staged surgery. Various authors have also explored the application of TID rods for severe scoliosis treatment. Kwong et al. [18] assessed patients who underwent posterior spinal fusion using a non-contoured titanium elastic nail on one side, performed in a single-stage surgery to achieve partial correction, while the contralateral side was instrumented, resulting in a 53.6% correction in the coronal plane. The authors suggested that temporary internal distraction in a single-stage setting could aid in gradually correcting severe pediatric spinal deformities during surgery. Although common, neuromonitoring alerts are in fact reversible [18]. In a separate study involving 51 patients with severe scoliosis [19], temporary internal distraction rods were utilized in the surgical treatment, leading to an average Cobb reduction of up to 81%, surpassing that of Halo traction, and this improvement was maintained over long-term follow-up. Despite more frequent intraoperative neuromonitoring changes, they were reversible and did not induce neurological deficits. Nevertheless, caution is advised, as significant corrections could occasionally result in delayed neurologic deficits despite intact neuromonitoring, and TID contributed to a shorter hospital stay [19]. Analyzing the results of their staged surgical intervention, the authors proposed that this method could potentially decrease the need for high-grade osteotomy (HGO) and high-risk osteotomies, such as three-column osteotomies, in correcting severe scoliosis [42]. Tan et al. [2] assessed patients with severe spinal deformities who underwent staged surgeries involving initial temporary internal distraction followed by posterior correction and instrumentation, showing a 59.7% total correction of the large coronal curve during the final follow-up. The mean correction of the primary sagittal angle was 48% at the final follow-up. Another study [19] indicated that the average Cobb angle decreased from 103° pre-surgery to 20° at final follow-up, with an intermediate angle of 55° in the staged procedures.

Changes in intraoperative neuromonitoring were identified in 25.4% of surgical cases. Following the reduction in deformity correction, an immediate enhancement was seen in the neuromonitoring assessments. Incidences of wound dehiscence were present in 5.8% of cases, deep infections in 9.7% of cases, screw slippage in 2% of cases, and delayed limb weakness in 2% of cases. Patient-reported outcomes experienced a significant improvement during the final follow-up assessment. In a separate investigation, temporary internal distraction rods led to a mean correction from a preoperative major curvature of 148° to an average of 79°, and the Cobb angle of kyphosis T5–T12, initially at 79°, was corrected to a mean of 59°. FVC% increased from 59.3% to 68.7%, while FEV1% rose from 61.4% to 71.3%. The average gain in stature was 6.7 cm, and rib prominence was rectified to 3–5 cm. By the time of the ultimate operation, the major curvatures were adjusted to an average of 55°, Cobb’s T5–T12 kyphosis to 35°, FVC% to 71.2%, FEV1% to 76.3%, height by a 3.1 cm increase, and rib prominence by a 1–3 cm correction. The mean time gap between the surgeries was 3.5 months. None of the patients exhibited postoperative neurological deficiencies or infections [40].

PF in severe idiopathic scoliosis patients was notably compromised in this investigation, potentially elevating the risks associated with corrective surgery and impacting the patients’ daily functionality, growth, development, and appearance [36,44]. Moreover, aligned with the outcomes of our analysis, extensive literature has established a robust linkage between curve correction and PF enhancement [5,12,14,25,32,44]: enhancing respiratory capacity before scoliosis correction may mitigate postoperative complications. Surgical interventions for severe scoliosis using HGT or staged procedures have substantially decreased apical deformity translation by 70% and bolstered postoperative PF in severe scoliosis cases [5,12,14,25,32,45,46]. The occurrence of restrictive pulmonary disorders in individuals with severe spinal deformities is also prevalent and could escalate morbidity and mortality rates [5,46,47]. In this study, the mean preoperative FVC predicted values (%) were markedly inferior in both cohorts compared with the final follow-up assessments. Pre-surgery, the mean FVC values stood at 54.5% in Group 1 and 49% in Group 2 (NS). Post-surgery and at the final follow-up, a statistically significant enhancement in values occurred for both groups (ranging from 25% to 56%). The mean FEV1 values were 60.8% in Group 1 and 58% in Group 2 (N.S.).

After definitive surgery and at final follow-up, these values also exhibited a statistically significant enhancement in both cohorts (ranging from 35% to 65%). These results suggest that employing either HGT or TID alongside staged surgery confers benefits in relation to PF within this particular group. Of paramount importance, the utilization of staged surgery for severe idiopathic scoliosis led to notable enhancements specific to the disease (as indicated by the outcomes of SRS-22r) over the course of the 2-year follow-up period. The documented mortality rate attributed to pulmonary complications in the management of intricate spinal deformities has underscored the critical need for thorough scrutiny and the meticulous preoperative preparation of high-risk patients to mitigate potential complications [5,12,14,32,36,44,45]. Within our investigation, surgical intervention played a pivotal role in enhancing the overall quality of life across various functional domains, including aspects related to aesthetics and the amelioration or eradication of bodily deformities among patients.

### Limitations

Our study, similar to most research endeavors, is constrained by certain limitations inherent to its retrospective design. Although the cohort of patients under investigation was limited in size, the recruitment of a substantial number of individuals with severe spinal curvatures proved to be challenging. However, it is fortunate that individuals afflicted with severe scoliosis represent a minority within the general population. Furthermore, as the primary objective of surgical intervention for spinal deformities is the prevention of severe scoliosis progression and the provision of surgical remedies for minor curvatures, an additional drawback of our study pertains to the inability to conduct a comparative analysis of the overall treatment costs associated with both approaches, a factor greatly influenced by the specific national context and healthcare insurance framework. The key strength of this investigation lies in its comprehensive examination of patients who underwent surgical procedures employing two distinct techniques within a uniform protocol and under the guidance of the same proficient spinal surgeon. The surgical interventions and technical parameters were also standardized across all cases to mitigate and obviate potential complications. Subsequent to the surgical interventions, all patients were subject to a follow-up period averaging 3 years. In conjunction with conventional radiographic and pulmonary function assessments, meticulous documentation and analysis of postoperative complications enabled the presentation of comprehensive pre- and postoperative data, alongside evaluations based on the PF and SRS-22r metrics, utilizing standardized performance indicators.

## 5. Conclusions

The surgical management of severe and neglected spinal deformities in pediatric and adolescent populations can be performed safely, with a tolerable incidence of minor complications. Alternative approaches, such as less invasive temporary internal distraction methods, may be considered in the management of severe spinal curvature disorders, offering potential advantages in terms of enhanced quality of life and respiratory function. Utilizing a less invasive temporary internal distraction technique followed by posterior spinal fusion yielded a substantial average correction of deformity, with 70% improvement in the coronal plane, 60% in the sagittal plane, and an average increase of 10.8 cm in trunk height. The post-treatment outcomes demonstrated a notable 76% reduction in rib hump deformity, along with enhancements in respiratory capacity, as evidenced by a 25–56% increase in predicted forced expiratory volume in 1 s and a 35–65% rise in forced vital capacity. These improvements culminated in a significant enhancement in quality of life, as measured by the SRS-22r scale, without necessitating more radical and high-risk interventions. The utilization of surgical interventions employing minimally invasive temporary internal distraction techniques has a considerable impact on the well-being of individuals suffering from severe scoliosis, contributing to enhanced overall functionality. However, it is important to note that the clinical and imaging results are the same in both cohorts, and each approach can be interchangeably utilized, depending on the individual patient selected.

## Figures and Tables

**Table 1:** Patient demographics.

	Group 1 (*n* = 20)	Group 2 (*n* = 42)	*p*
Sex			
Male	2	10	NS
Female	18	32	NS
Mean age at surgery, years (SD)	16.5 (3.5)	16.4 (4.8)	NS
Mean (SD) follow-up, years	3.8 (1.8)	4.1 (1.8)	NS
Mean BMI at surgery (SD)	23.2 (5.8)	23.6 (5.9)	NS
Mean amount of segment involvement fusion (SD)	12.5 (2.5)	13 (3.5)	NS
Percentage (*n*) of patients fused below L3	68%	61%	NS
Mean duration/stay at hospital, days (SD)	42.5 (8.8)	First stage: 6 (2.1) Second stage: 4 (1.2)	
Mean duration of surgery, min (SD)	HGT: 28 (14.5) Final surgery: 352 (78.5)	First stage: 189.5 (44.9) Second stage: 264 (69.6)	
Mean blood loss at surgery, mL (SD)	588 (288)	First stage: 282 (42) Second stage: 458 (245)	
Mean total HGT duration, days (SD)	36 (6.9)	NA	

NA means not applicable; NS means not statistically significant.

**Table 2:** Radiological parameters before surgical treatment (pre- and postoperative) and at final follow-up.

	Group 1 (*n* = 20)	Group 2 (*n* = 42)	*p*-Value (G1 vs. G2)
Mean (SD) preoperative Cobb, °	124 (10.8)	122 (9.8)	0.426
Mean (SD) Cobb after initial distraction (Halo, LITID), °	76.4 (28.8)	68 (18.2)	<0.001
Mean (SD) Cobb after definitive fusion, °	45 (13.8)	37.4 (11.4)	<0.001
Mean (SD) Cobb at final follow-up, °	44.9 (12.2)	40.9 (11.8)	0.738
*p*-Value (preop vs. final follow-up)	<0.001	<0.001	
Mean (SD) major preoperative thoracic kyphosis, °	96.5 (14.5)	92 (11.8)	0.782
Mean (SD) major thoracic kyphosis after initial distraction (Halo, LITID), °	68.5 (21.2)	61 (16.6)	<0.001
Mean (SD) major thoracic kyphosis after definitive fusion, °	45.8 (15.8)	36.2 (10.2)	<0.001
Mean (SD) major thoracic kyphosis at final follow-up, °	44 (15.8)	34.3(8.6)	0.298
*p*-Value (preop vs. final follow-up)	<0.001	<0.001	
Mean (SD) preoperative lumbar lordosis T12–S1, °	−60.6 (23.6)	−46 (10.8)	<0.001
Mean (SD) lumbar lordosis T12–S1 after initial distraction (Halo, LITID), °	−57.5 (27.4)	−41 (8.9)	<0.001
Mean (SD) lumbar lordosis T12–S1 after definitive fusion, °	−41 (12.9)	−36.4 (10.6)	<0.001
Mean (SD) lumbar lordosis T12–S1 at final follow-up, deg°	−48.2 (13.5)	−42.4 (8.9)	0.178
*p*-Value (preop vs. final follow-up)	<0.001	0.142	
Mean (SD) preoperative apical vertebral translation, mm	78 (21.8)	73.2 (21.5)	0.492
Mean (SD) apical vertebral translation after initial distraction (Halo, LITID), mm	54.4 (26.2)	54.2 (24.2)	0.757
Mean (SD) apical vertebral translation after definitive fusion, mm	33.8 (17.6)	28.6 (16.8)	0.362
Mean (SD) apical vertebral translation at final follow-up, mm	35 (14.6)	28.2 (18.4)	0.393
*p*-Value (preop vs. final follow-up)	<0.001	<0.001	

**Table 3:** Pulmonary function and thoracic parameters at pre- and postoperative and final follow-up.

	Group 1 (*n* = 20)	Group 2 (*n* = 42)	*p*-Value
Mean (SD) preoperative forced vital capacity, percentage of predicted	54.5 (18.9)	49 (16.2)	0.132
Mean (SD) forced vital capacity after initial distraction (Halo, LITID), percentage of predicted	66.7 (15.9)	55.2 (16.8)	<0.001
Mean (SD) forced vital capacity after definitive fusion, percentage of predicted	73.4 (7.2)	67 (10.2)	<0.001
Mean (SD) forced vital capacity, percentage of predicted at final follow-up	74.9 (12.2)	76 (13.2)	0.272
*p*-Value (preop vs. final follow-up)	<0.001	<0.001	
Mean (SD) preoperative forced expiratory volume in 1 s, percentage of predicted	60.8 (13.9)	58 (12.8)	0.221
Mean (SD) forced expiratory volume in 1 s after initial distraction (Halo, LITID), percentage of predicted	70.1 (10.8)	66 (13.9)	<0.001
Mean (SD) forced expiratory volume in 1 s after definitive fusion, percentage of predicted	74.4 (14.8)	71.2 (16.2)	0.862
Mean (SD) forced expiratory volume in 1 s, percentage of predicted at final follow-up	75.9 (12.5)	78 (11.8)	0.964
*p*-Value (preop vs. final follow-up)	<0.001	<0.001	
Mean (SD) preoperative rib hump difference, cm	8.6 (2.4)	8.2 (1.9)	0.881
Mean (SD) rib hump difference after definitive fusion, cm	2.8 (1.9)	2.2 (1.32)	<0.001
Mean (SD) rib hump difference at final follow-up, cm	2.4 (1.8)	1.98 (1.7)	0.911
*p*-Value (preop vs. final follow-up)	<0.001	<0.001	
Mean (SD) preoperative trunk height difference, cm	28.8 (7.1)	29.2 (4.2)	0.887
Mean (SD) trunk height difference after definitive fusion, cm	36.5 (6.8)	38.3 (2.8)	<0.001
Mean (SD) trunk height difference at final follow-up, cm	37.2 (8.2)	40 (3.2)	0.829
*p*-Value (preop vs. final follow-up)	<0.001	<0.001	

**Table 4:** SRS-22R scores in the surgical study groups.

SRS-22R	Group 2 (*n* = 42) LITID		Group 1 (*n* = 20) HGT		LITID	HGT	LITID vs. HGT
Parameter	Preoperative (A)	Final Follow-Up (B)	Preoperative (C)	Final Follow-Up (D)	*p*-Value A vs. B	*p*-Value C vs. D	*p*-Value B vs. D
Function	3.30 (0.42)	4.42 (0.42)	3.10 (1.22)	4.48 (0.66)	0.11	0.141	0.92
Pain	3.22 (0.25)	3.88 (0.70)	2.82 (1.16)	4.08 (0.78)	0.129	<0.001	0.87
Self-image	3.16 (0.68)	3.98 (0.78)	3.12 (0.88)	4.42 (0.66)	<0.001	<0.001	0.93
Mental health	2.92 (0.62)	4.32 (0.90)	2.88 (0.86)	4.28 (0.70)	<0.001	<0.001	0.86
Satisfaction	2.80 (0.86)	4.42 (0.56)	2.50 (0.88)	4.40 (0.60)	<0.001	<0.001	0.93
Total score	3.22 (0.42)	4.46 (0.45)	2.88 (0.92)	4.33 (0.65)	<0.001	<0.001	0.78

**Table 5:** Rate of complications during the course of the treatment.

Complication Rates Following Posterior Final Fusion	Group 2 (*n* = 42) LITID	Group 1 (*n* = 20) HGT	*p*
Intraoperative neuromonitoring changes	5 (12%)	3 (15%)	NS
Superficial wound infection	2 (5%)	1 (5%)	NS
Pneumonia	2 (5%)	2 (10%)	NS
Paresthesia from the lateral cutaneous nerve of the lower limb	5 (12%)	3 (15%)	NS
Pin infections	NA	7 (35%)	NS
Deep infection	1 (2%)	1 (5%)	NS
SMAS	2 (5%)	1 (5%)	NS
Neck/back pain during traction/temporary internal distraction	3 (7%)	11 (55%)	<0.001
Total	20 (48%)	28 (140%)	<0.001

NA means not applicable; NS means not statistically significant.

## Data Availability

The data are contained within the article.
